# Using patient-reported data from a smartphone app to capture and characterize real-time patient-reported flares in rheumatoid arthritis

**DOI:** 10.1093/rap/rkac021

**Published:** 2022-03-16

**Authors:** Julie Gandrup, David A Selby, Sabine N van der Veer, John Mcbeth, William G Dixon

**Affiliations:** 1 Centre for Epidemiology Versus Arthritis, Division of Musculoskeletal and Dermatological Sciences; 2 Centre for Health Informatics, Division of Informatics, Imaging and Data Sciences, University of Manchester, Manchester; 3 Department of Rheumatology, Salford Royal NHS Foundation Trust, Salford, UK

**Keywords:** RA, flare, patient-generated health data, mHealth, smartphone

## Abstract

**Objective:**

We aimed to explore the frequency of self-reported flares and their association with preceding symptoms collected through a smartphone app by people with RA.

**Methods:**

We used data from the Remote Monitoring of RA study, in which patients tracked their daily symptoms and weekly flares on an app. We summarized the number of self-reported flare weeks. For each week preceding a flare question, we calculated three summary features for daily symptoms: mean, variability and slope. Mixed effects logistic regression models quantified associations between flare weeks and symptom summary features. Pain was used as an example symptom for multivariate modelling.

**Results:**

Twenty patients tracked their symptoms for a median of 81 days (interquartile range 80, 82). Fifteen of 20 participants reported at least one flare week, adding up to 54 flare weeks out of 198 participant weeks in total. Univariate mixed effects models showed that higher mean and steeper upward slopes in symptom scores in the week preceding the flare increased the likelihood of flare occurrence, but the association with variability was less strong. Multivariate modelling showed that for pain, mean scores and variability were associated with higher odds of flare, with odds ratios 1.83 (95% CI, 1.15, 2.97) and 3.12 (95% CI, 1.07, 9.13), respectively.

**Conclusion:**

Our study suggests that patient-reported flares are common and are associated with higher daily RA symptom scores in the preceding week. Enabling patients to collect daily symptom data on their smartphones might, ultimately, facilitate prediction and more timely management of imminent flares.

Key messages

- Patient-reported flares were common, occurring at least once in 75% of RA patients over 3 months.
- Patient-reported flares were associated with higher mean scores in daily RA symptoms in the preceding week.
- Frequent patient-reported data might, ultimately, facilitate prediction and more timely management of RA flares.

## Introduction

Treatment of patients with RA aims to control disease activity and sustain remission [1]. Although major advancements in the treatment of RA have made these realistic goals for many patients [2], RA patients (even those in remission) still experience transient episodes of worsening disease activity called flares [3, 4]. These fluctuations in disease activity are associated with poor clinical outcomes, can lead to progression of radiographic joint damage and impaired function, and accelerate cardiovascular co-morbidity [5–8]. Suboptimal management of flares remains a hurdle in optimizing outcomes, including quality of life and activities of daily living, for people living with RA, despite the availability of more effective treatments and treat-to-target approaches.

To date, most studies of RA flares have defined flares using patient recall at infrequent intervals, usually 3–12 months apart [9, 10]. These methods can result in missing flares owing to recall error and therefore lead to an underestimation of the real prevalence of flares in RA. In routine clinical care, flares occurring between scheduled consultations might also not be captured by commonly used disease activity measures, such as the DAS28. This incomplete information about flares leads to delayed and missed treatment opportunities, which, in turn, can have a negative effect on patient outcomes. This implies an unmet need to capture and explore transient flares with greater accuracy. The same is true for RA symptoms more broadly, and capturing these alongside self-reported flares might provide new insights into the temporal relationship between them.

With the increasing adoption of smartphones and use of digital technology in clinical care and research, we now have an opportunity to collect health data directly from patients and at higher frequency. These technologies make it possible to capture and characterize day-to-day variations in disease severity and occurrence of flares in real time, instead of relying on patient recall at the discrete intervals of traditional research in cohorts and registers or at infrequent clinical appointments. This opportunity of better characterizing day-to-day changes and acute deterioration in disease expands way beyond RA into other rheumatic and long-term disease areas, such as mental health and oncology [11, 12].

In this study, we aimed to characterize patient-reported flares using daily symptom data collected through a smartphone app in people living with RA. Specific objectives were to understand the frequency and duration of patient-reported flares and to explore associations between symptom summary features and patient-reported flares.

## Methods

We followed the Strengthening the Reporting of Observational Studies in Epidemiology (STROBE) checklist for reporting this study [13].

### Setting and participants

We conducted a secondary analysis of patient-reported symptom data obtained for the REmote MOnitoring of RA (REMORA) study [14]. The primary aim of the REMORA study was to test the feasibility of collecting daily patient-reported symptoms from 20 RA patients over 85 days using a smartphone app, with data integrated into the electronic health record. Patients were recruited from the rheumatology outpatient clinic at a single hospital site (Salford Royal NHS Foundation Trust, UK) in 2016. Patients were eligible if they had clinician-verified RA and were willing to participate and able to provide written consent. They could have either active or inactive disease. After consenting, members of the research team set up patients’ phones, provided user instructions verbally and supported them throughout the study.

**Fig. 1:**
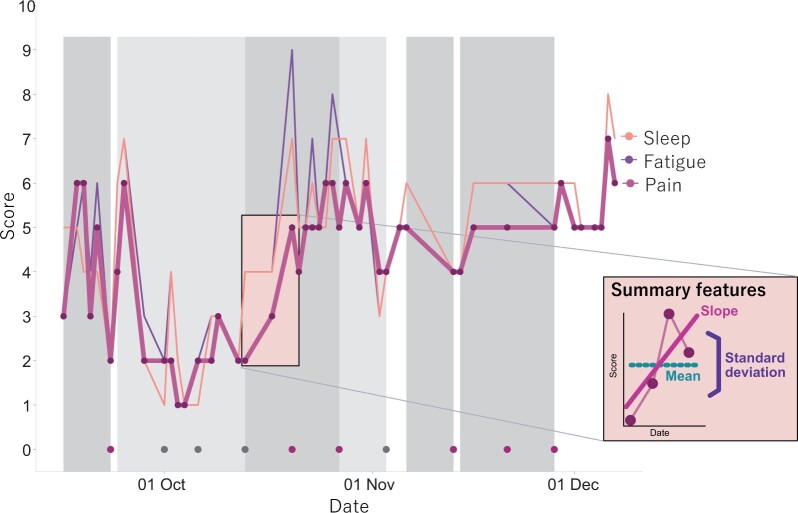
Example of raw daily and weekly data Example patient illustrating symptom tracking for three (of seven) selected daily symptoms and weekly flares. The red dots towards the bottom indicate that the patient answered ‘yes’ to the weekly flare question, the grey dots when the patient answered ‘no’. Missing flare reports are not represented here. The 7 days leading up to the flare question are highlighted as either a flare week (darker grey) or a non-flare week (lighter grey). The inset in the lower right corner explains the three summary features for pain as a symptom: mean, standard deviation (variability) and slope.

All patients were prompted to enter seven daily symptoms on a 0–10 numerical rating scale (NRS), where 10 represented the highest symptom severity. Items were adapted from the RA Impact of Disease questionnaire for daily use [15] (see Table 1 for a list of data items relevant to this analysis). Once a week, patients were asked if they had experienced a flare in the preceding week. Patients could view their own data as graphs over time in the app, but data were not reviewed by the clinical team in between clinical appointments, and patients were advised to take the usual action in case of health problems. During a subsequent clinical research consultation that mimicked a typical consultation, patients and clinicians reviewed the data in the electronic health record together. All 20 patients and their daily and weekly patient-reported data were included in this analysis. An illustration of a single patient’s tracked symptoms and self-reported flares is shown in Fig. 1.

**Table 1:** Daily and weekly data items collected on the REMORA app included in this analysis

Item	Prompt	Scale	Range
Daily			
Pain	Select the number that best describes the pain you felt due to your RA during the last 24 h	NRS	None, 0; extreme (10)
Function	Select the number that best describes the difficulty you had in doing daily physical activities due to your RA during the last 24 h	NRS	No difficulty, 0; extreme difficulty (10)
Fatigue	Select the number that best describes how much fatigue you felt due to your RA during the last 24 h	NRS	No fatigue, 0; totally exhausted (10)
Sleep	Select the number that best describes the sleep difficulties (i.e. resting at night) you felt due to your RA during the last 24 h	NRS	No difficulty, 0; extreme difficulty (10)
Physical well-being	Considering your arthritis overall, how would you rate your level of physical well-being during the last 24 h?	NRS	Very good, 0; very bad (10)
Emotional well-being	Considering your arthritis overall, how would you rate your level of emotional well-being during the last 24 h	NRS	Very good, 0; very bad (10)
Coping	Considering your arthritis overall, how well did you cope (manage, deal, make do) with your RA during the last 24 h?	NRS	Very well, 0; very poorly (10)
Weekly			
Occurrence of flare	Have you experienced a flare in the last week?	Dichotomous	Yes; No

NRS: Numerical rating scale.

### Definition of flares and explanatory variables

#### Patient-reported flares

The occurrence of patient-reported flares was used as the outcome, which was derived from the weekly question prompted via the app every seventh day. The question ‘Have you experienced a flare in the last week?’ could be answered ‘yes’ or ‘no’. What classified as a flare was left to the discretion of the patient answering the question. The 7 days before the weekly flare question were deemed to be a flare week if the patient answered ‘yes’. Conversely, if the patient answered ‘no’ the week was deemed a non-flare week. Weeks with missing flare data (i.e. an unanswered flare question) were not included in the analysis.

Owing to the way in which the app was configured, it was possible for patients to answer the weekly flare question at their own instigation outside of the prompted weekly schedule. To deal with answers to non-scheduled flare questions, we set up the following two rules: if patients answered the flare question more than once on the same day, we kept the entry with a flare if the multiple responses differed; and if patients answered the flare question on consecutive days or days closer than 5 days of each other, we kept the entry that was closest to the original 7-day scheduled questions or the earliest entry in that week if none fitted the weekly pattern.

#### Symptom summary features

For each week before the flare question, we calculated the following symptom summary features across the daily symptoms in that week as our explanatory variables: mean score, s.d**.** and slope (see Fig. 1). The mean score represented symptom severity. The s.d**.** was chosen as a measure of variability of the symptoms in the preceding week. It is the most common measure of variability, which averages the absolute deviation of the symptom score (e.g. pain) of each day from the mean over the 7-day period, thus capturing symptom volatility. The slope was equal to the beta coefficient from fitting a linear model through the daily data points of the preceding week, thus capturing both the extent of change and the change direction (i.e. positive or negative). The patient-reported symptom scores were ordinal variables, but for the purpose of this analysis they were treated as continuous variables.

In preparation for modelling (see below under “Associations between patient-reported symptoms and flares”), we explored correlations between the summary features of symptoms with a correlation plot calculating Pearson’s correlation coefficients for combinations of symptom summary features.

### Statistical analysis

We used descriptive statistics to summarize patient age, gender and ethnicity [categorical variables as count (percentage) and continuous variables as median (interquartile range, IQR)].

Each patient’s time in the study was calculated as the number of days between first and last active symptom reporting, with a maximum of 85 days. We calculated completion rates for daily and weekly questions. For daily entries, the numerator was the number of days on which at least one symptom score was completed, with the denominator as the patient’s time in the study. For weekly entries, the numerator was the number of completed weekly responses, and the denominator was the number of weeks in which a weekly question set was triggered.

#### Frequency and duration of flares

For flare frequency, we calculated the proportion of patients reporting at least one flare over the course of the study. For flare duration, we counted the number of consecutive weeks patients reported flares.

#### Descriptive comparison of symptom summary features between flare and non-flare weeks

We calculated summary means of the symptom summary features in flare and non-flare weeks. We looked at the mean symptom scores in a patient’s flare weeks and compared that with the mean symptom score in the patient’s non-flare weeks, and then averaged across the population. The same comparison was made for the other two symptom summary features: s.d. and slope.

#### Associations between patient-reported symptoms and flares

For modelling purposes, we only included participant weeks that had ≥5 days of daily symptom data before a completed flare question (answering either ‘yes’ or ‘no’). This was to ensure a balance between excluding too many participant weeks and the possibility of daily data missing not at random. To assess the impact of different definitions of a participant week on our findings, we performed two sensitivity analyses including participant weeks having 7 days of daily symptom data (i.e. complete weeks) and participant weeks with ≥1 day of daily entries (i.e. all weeks).

To quantify the associations between patient-reported flares and the seven daily symptoms, we used mixed effect logistic regression analyses, with patients as the random effect, which took into account the hierarchical structure of the data with multiple measurements within patients. The analyses were performed with flare week yes/no as a binary dependent variable. The three symptom summary features were used as explanatory variables. The modelling followed a two-step approach: first, univariate modelling looked at the derived summary features of one symptom at a time in its own model, resulting in 21 distinctive models (three symptom summary features across each of the seven daily symptoms), followed by multivariate modelling wherein we included all three summary features for a specific symptom (resulting in seven models: one for each symptom). We initially considered one model that included the three summary features and all seven symptoms simultaneously, but this was not possible owing to strong collinearity between individual symptoms (see Supplementary Fig. S1, available at *Rheumatology Advances in Practice* online). For all models, we reported unadjusted odds ratio (OR) estimates with 95% CI. All analyses were performed in R v.4.0.5 (R Core Team, 2021) [16].

## Results

Twenty RA patients took part in the study, of whom 14 were female (70%). The median age was 58.5 (IQR 48, 64) years, and all except one (95%) were of white British ethnicity. The median number of days in the study was 81 (IQR 80, 82). A total of 9177 daily symptom scores were submitted through the app out of 11 011 possible entries (i.e. an 83% completion rate). A total of 198 weekly flare questions were answered throughout the study period out of a possible 225 weeks, resulting in a completion rate of 88%. Fig. 1 shows an example of raw daily and weekly symptom tracking data for one patient in the cohort.

### Frequency and duration of patient-reported flares

**Fig. 2:**
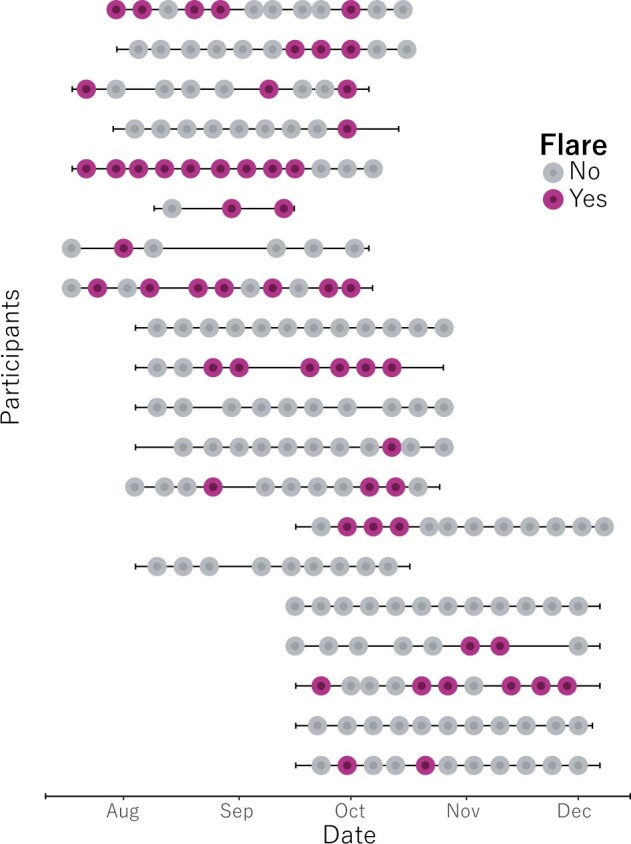
Overview of flare distribution for each patient in the REMORA study A pink dot indicates that the patient answered ‘yes’ to the weekly flare question in the REMORA app. A grey dot indicates a ‘no’ answer. Horizontal lines represent the time from first tracked symptom to last (i.e. time in study for each patient).

Fifteen of 20 patients (75%) reported at least one flare week over the 3-month study period, with 54 patient-reported flares in total out of 198 answered flare questions. Patients reported a median of two flare weeks (IQR 0.5–4). Fig. 2 shows that, of the patients reporting a flare, two-thirds (10 of 15) reported flares for two or more consecutive weeks, and one-third (5 of 15) reported flares for three or more consecutive weeks.

### Descriptive comparison of symptom summary features between flare and non-flare weeks

All mean symptom scores were higher [difference on average 0.67 (s.e. 0.24)] in flare weeks compared with non-flare weeks (Table 2). The s.d., a measure of variability, was marginally higher in flare weeks. For slope, there was a small but consistently positive increase for all symptoms in flare weeks.

**Table 2:** Differences in mean symptom summary features (mean, s.d. and slope) across seven daily symptoms in flare and non-flare weeks

Symptom summary feature	Symptom	**Flare weeks (*n*** ^a^ **= 15)**	**Non-flare weeks (*n*** ^a^ ** = 15)**
Mean (s.d.) symptom score in the week before flare reporting	Pain	4.4 (1.8)	3.6 (1.9)
	Function	4.2 (1.7)	3.4 (2.0)
	Fatigue	4.4 (2.0)	3.8 (1.8)
	Sleep	4.1 (2.5)	3.9 (2.4)
	Emotional well-being	3.9 (1.6)	3.3 (1.5)
	Physical well-being	4.2 (1.5)	3.3 (1.6)
	Coping	3.9 (1.4)	3.1 (1.5)
Standard deviation (s.d.) of symptom scores in the week before flare reporting	Pain	1.0 (0.4)	0.7 (0.4)
	Function	0.9 (0.4)	0.8 (0.4)
	Fatigue	1.0 (0.5)	0.8 (0.4)
	Sleep	0.8 (0.5)	0.9 (0.5)
	Emotional well-being	0.9 (0.5)	0.7 (0.3)
	Physical well-being	1.0 (0.4)	0.7 (0.3)
	Coping	0.9 (0.4)	0.7 (0.5)
Slope (s.d.) of symptom scores in the week before flare reporting	Pain	0.12 (0.19)	−0.01 (0.14)
	Function	0.09 (0.17)	−0.02 (0.13)
	Fatigue	0.08 (0.23)	−0.04 (0.12)
	Sleep	0.10 (0.17)	−0.01 (0.18)
	Emotional well-being	0.06 (0.19)	−0.05 (0.14)
	Physical well-being	0.13 (0.17)	0.02 (0.14)
	Coping	0.10 (0.15)	−0.06 (0.15)

a *n* refers to the number of participants contributing data to the analysis.

### Associations between daily symptoms and flares

**Fig. 3:**
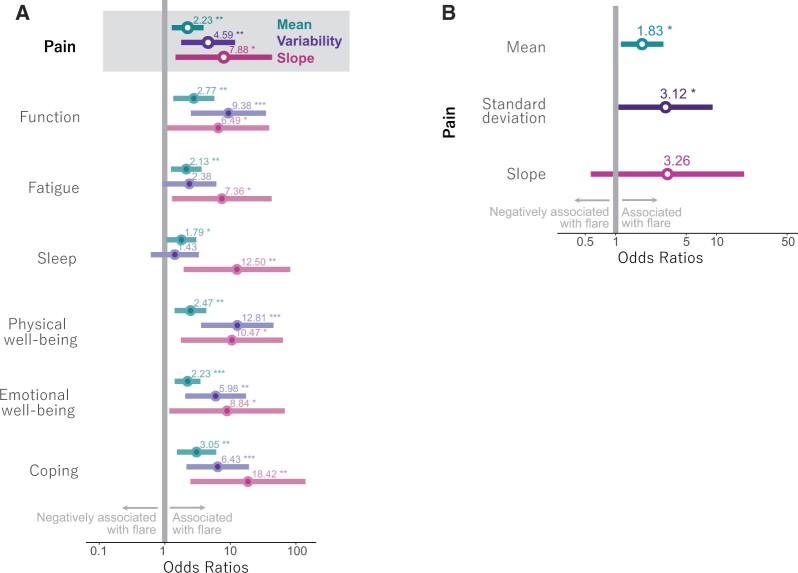
Associations between summary features and flare state (**A**) Univariate mixed effect logistic regression modelling showing, for each symptom, the associations between three symptom summary features (mean, s.d./ variability and slope) and flare state. (**B**) Multivariate modelling of pain using each of the three symptom summary features.

Daily symptoms were reported on ≥5 days for 168 of 198 weeks in which a flare question was answered. Univariate modelling of data from these 168 participant weeks revealed that flare occurrence was significantly associated with higher mean scores across all seven symptoms (Fig. 3A). For instance, a single unit increase in mean pain score over the week was associated with a twofold increased likelihood of a flare [OR 2.23 (95% CI 1.28, 3.90)]. Likewise, higher s.d**.** of all symptoms except fatigue and sleep was significantly associated with flare occurrence, but the 95% CIs were wide. Larger slopes (i.e. more steeply increasing scores) of all symptoms were also significantly associated with occurrence of flares, although also here the confidence intervals were wide.

Fig. 3B shows that, in the multivariate model for pain using each of its three derived symptom summary features, mean pain scores appeared to be more clearly associated with a flare [OR 1.83 (95% CI 1.15, 2.97)] than the change in scores in the preceding week [OR 3.26 (95% CI 0.57, 18.74) for slope]. Variability was also significantly associated with higher odds of flares [OR 3.12 (95% CI 1.07, 9.13) for s.d.], but with a wider CI. Multivariate models for the remaining six symptoms showed comparable significant results for mean scores, with ORs ranging between 1.64 and 2.13. Likewise, associations with s.d. and slope were less convincing, with wide CIs (Supplementary Fig. S2, available at *Rheumatology Advances in Practice* online).

### Sensitivity analyses

Sensitivity analyses for univariate models with two different definitions of a participant week showed similar results: when running the models using the complete weeks (*n* = 88 participant weeks) definition and the all weeks definition (*n* = 198 participant weeks), we found that higher scores of the majority of symptoms were still significantly associated with an increased likelihood of flare occurrence (Supplementary Fig. S3, available at *Rheumatology Advances in Practice* online).

When running the multivariate pain model, mean pain remained significantly associated with higher odds of flare occurrence for both definitions. When looking at the broadest definition of a participant week (all weeks), the association with s.d. was no longer as clear (Supplementary Table S1, available at *Rheumatology Advances in Practice* online).

## Discussion

This study demonstrated the ability to use real-time daily patient-reported symptom data to characterize patient-reported flares in RA. We showed that self-reported flares were frequent, occurring in 75% of patients over 3 months. The majority of patients experienced more than one flare. Patients had higher scores (for mean, variability and slope) across a range of daily symptoms in the week preceding a flare. When looking at the relative importance of daily symptom summary features on the occurrence of flares, higher mean scores in the week preceding the flare seemed more important for the likelihood of a flare occurring compared with symptom variability and slope; it matters more to have higher symptom scores rather than varying or increasing scores.

We found that 75% of patients reported to have experienced a flare over the 3-month study period, and the majority reported more than one flare. In a cohort of Danish RA patients in remission or low disease activity at baseline, Kuettel *et al.* [17] found a prevalence of self-reported flares of 36% when asked ‘Are you experiencing a flare of your RA at this time?’ at 3-month intervals. These proportions were slightly lower than an observational study in established RA, where the frequency of self-reported flares (‘During the past 6 months, have you had a flare in your rheumatoid arthritis?’) ranged from 54 to 74% when asked at 6-month intervals [18]. Despite different anchor questions to detect flares, various periods of recall and differences in RA patient populations (unselected disease *vs* remission/low disease activity *vs* established RA), previous work and our study underline that self-reported flares are common in RA patients.

We defined a flare from the patient’s perspective. The weekly flare question used here was developed for the REMORA study and has not been validated externally. Currently available and validated flare measurement tools (such as the OMERACT Flare Questionnaire and the FLARE-RA questionnaires [10, 19]) do not allow for simple, one-item weekly sampling, hence our flare question was intentionally pragmatic. With this simple question, the term flare was left open to interpretation by patients. This approach is likely to have yielded a range of flare experiences and intensities. The concept of flares and its definition usually differ according to patient and clinician views: patients can focus on subjective changes, such as pain, general signs, mood disturbance and the need to seek help [3], whereas clinicians are more likely to consider objective changes, such as tender and swollen joint counts or increased inflammatory markers, on which they can base treatment decision-making [9]. However, patient-generated health data are increasingly acknowledged as an important aspect of managing patients with RA, especially given an acceleration of virtual care during the COVID-19 pandemic, justifying a patient-centric approach [20].

We chose mean (s.d.) and slope as our symptom summary features because they capture different aspects of the symptom data in the week preceding a flare and have been reported in other studies in different musculoskeletal conditions [21, 22]. They are intuitive and interpretable; higher/lower scores, higher/lower variability in scores and steep/gradual increase or decrease in scores. In our analyses, the mean showed the clearest association with the occurrence of flare across all models. A cautious interpretation would be that, in our cohort, flares seem to be particularly driven by higher mean scores. For pain, we also found that even a modest change in mean score increased the likelihood of a flare [OR 2.23 (95% CI 1.28, 3.90) for the univariate model]. To contextualize this number, a 15% change in pain is considered to be a clinically important difference in RA [23], highlighting the clinical utility of using daily symptoms to identify meaningful deteriorations. Owing to our small sample size, we were limited in how detailed the exploration of the associations with flares could be. Larger datasets would allow for more sophisticated methods for summarizing daily data and could shed more light on these associations. This would, however, need to be balanced against easy interpretability.

In the future, frequent self-monitoring of common symptoms using digital devices could aid in the early detection, even prediction, of flares and deteriorations in clinical settings. These data could be used to alert a clinician or clinical team, opening up opportunities to intervene and prevent, even in patients in otherwise stable remission. Such just-in-time interventions might include self-management advice, treatment adaptations or triggering a clinical contact. One early-stage study, so far reported as an abstract, explored classification of patient-reported flares using patient-reported outcomes collected on a smartphone app [24]. They found that daily pain scores and specific individual items from the OMERACT FLARE Instrument appeared effective in classifying new-onset flares, confirming the early feasibility demonstrated by our study of using frequently collected patient-reported measures to predict flares. Some qualitative studies have raised concerns about patients feeling reminded about their disease when doing frequent symptom tracking, resulting in either making patients too preoccupied with their disease or an internal resistance to use the app [25, 26]. Additionally, mHealth studies are inherently vulnerable to high attrition rates. Although the REMORA study saw high engagement throughout the study period (for more details, see Austin *et al.* [14]), approaches for maximizing engagement with symptom tracking need to be considered actively [27]. Exploring the use of passive sensor data as a proxy for patient-reported flares is another interesting development that would alleviate the patient burden of manually entering data with high frequency [28]. Translating such results into clinical care models, however, requires careful implementation including validation and clinical acceptability.

### Limitations

There are a number of limitations to our study. First of all, this was a pilot study, with few participants from a selected group of patients in one clinic, potentially limiting the generalizability of our results. Laboratory data, such as CRP, or disease activity measures, such as the DAS28 or the Clinical Disease Activity Index (CDAI), were not collected, preventing us from examining the relationship between patient-reported flares and established composite measures of disease activity. A prospective study linking patient-reported symptoms and flares with frequent clinically reported disease activity measurements would address this shortcoming. Additionally, we did not have access to information about treatment, medications and self-management strategies, which would have contextualized our results further.

Finally, the high correlation between the daily symptoms in combination with the limited sample size hampered the development of a full, multivariate model to quantify which symptom or summary feature (or combination within and across these) had the strongest association with flares. A future study with a larger sample size would allow us to start developing flare prediction models, in which dimensionality reduction techniques could be applied to account for the high correlation.

### Conclusion

In our RA cohort, self-reported flares were frequent. Flare weeks were broadly associated with higher scores (for mean, variability and slope) across a range of daily symptoms in the preceding week. When looking at associations between symptom summary features and patient-reported flares, the mean score showed the clearest association with the occurrence of flare across all seven common symptoms examined. For variability and slope, the association was less conclusive, largely owing to the limited sample size.

Our study is an early example of what daily changes in RA symptoms and prospectively collected self-reported flares might look like. Future analysis of daily symptoms might allow us to predict imminent flares, opening the opportunity for just-in-time interventions.

## Supplementary Material

rkac021_Supplementary_Data
