# Supplementary material for: Using patient-reported data from a smartphone app to capture and characterize real-time patient-reported flares in rheumatoid arthritis
Source: Rheumatol Adv Pract. 2022 Mar 16;6(1):rkac021. doi: 10.1093/rap/rkac021 (PMC8982773; doi:10.1093/rap/rkac021)
Supplement: rkac021_Supplementary_Data [file rkac021_supplementary_data.docx]

Supplementary material

**Supplementary Figure S1**: Correlation plot (Pearson’s correlation coefficients) of A**.** Daily symptoms scores and **B.** Symptom summary features

**
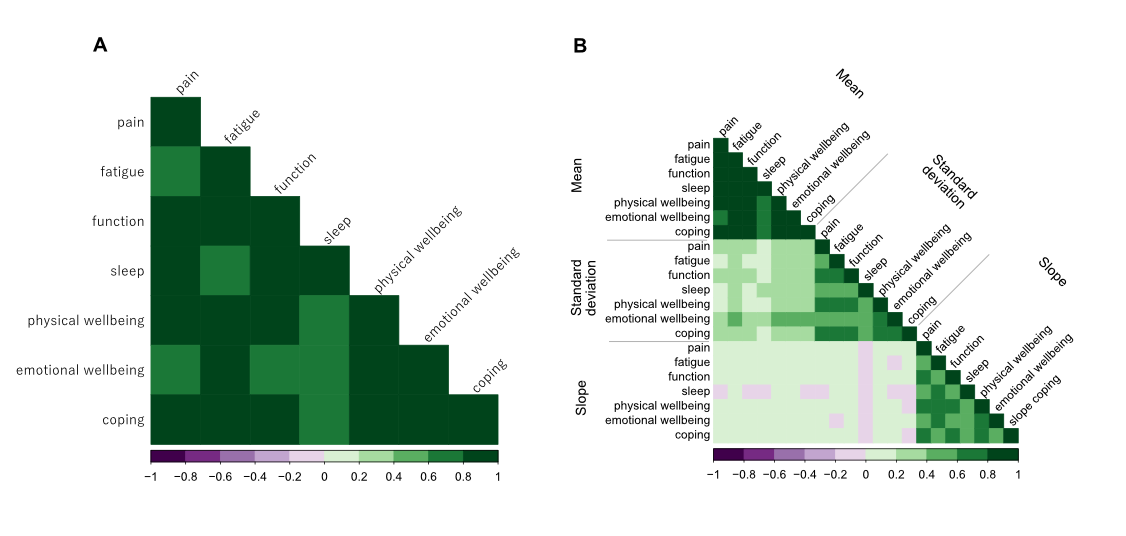
**

**Supplementary Figure S2:** Multivariate mixed effect logistic regression models for six daily symptoms and each of their three symptom summary features exploring the effects on patient-reported flares.

**
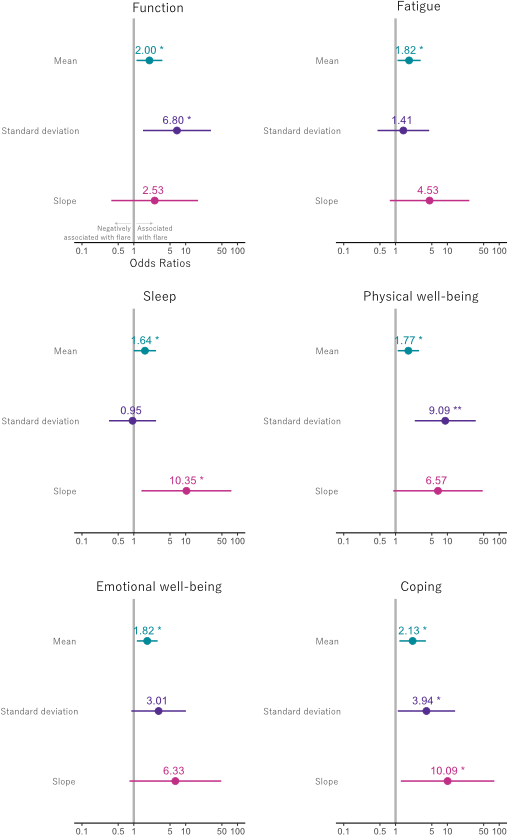
**

**Supplementary Figure S3:** Sensitivity analyses of different definitions of a participant week for univariate modelling. A: All weeks (no restrictions on days of daily data in a participant week, n=198). **B**: Complete weeks (only 7 days of daily data in a participant week, n=88).


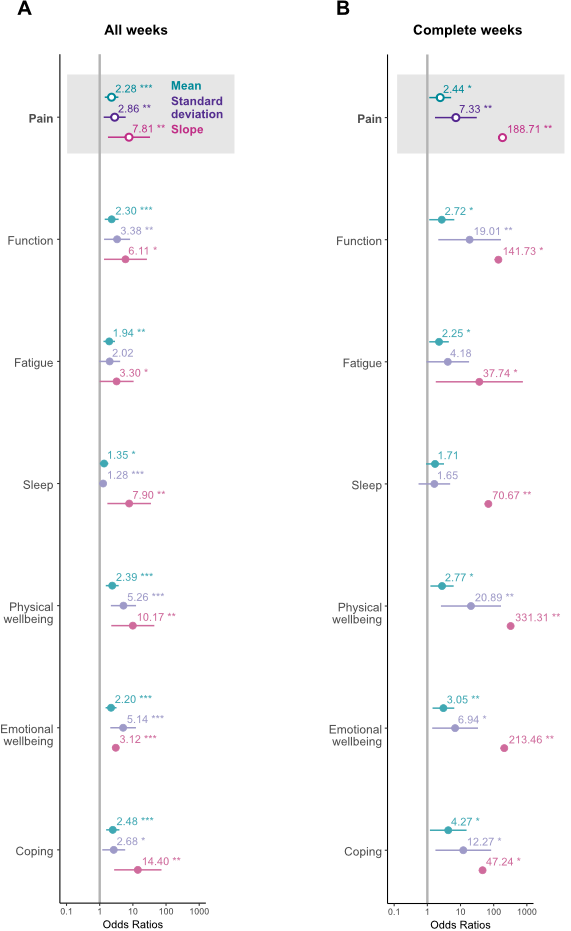


**Supplementary Table S1:** Results from sensitivity analyses of different definitions of a participant week for multivariate analyses looking at associations between symptom summary features of pain and the occurrence of patient-reported flare. “All weeks” is the broadest definition, including all participant weeks no matter the number of daily reporting in that week. “Complete weeks” is the most restrictive definition, only including participant weeks with seven days of daily data.

|  | OR (95% CI) | P value* |
| --- | --- | --- |
| **5-7 days (n=168)** | | |
| Mean pain | 1.83 (1.15-2.97) | **< 0.05** |
| SD pain | 3.12 (1.07-9.13) | **< 0.05** |
| Slope pain | 3.26 (0.57-18.74) | 0.19 |
| **All weeks (n=198)** | | |
| Mean pain | 1.94 (1.25-2.30) | **<0.01** |
| SD pain | 1.99 (0.83-4.76) | 0.12 |
| Slope pain | 3.71 (0.82-16.85) | 0.09 |
| **Complete weeks (n=88)** | | |
| Mean pain | 1.89 (1.03-3.48) | **<0.05** |
| SD pain | 4.50 (0.88-28.44) | 0.07 |
| Slope pain | 30.36 (0.80-1146.58) | 0.07 |

OR = odds ratio, CI = confidence interval

* Modelling results calculated via multi-level mixed effects logistic regression modelling
